# The safety of early administration of oral fluid following general anesthesia in children undergoing tonsillectomy: a prospective randomized controlled trial

**DOI:** 10.1186/s12871-020-01230-4

**Published:** 2021-01-11

**Authors:** Meng-Hang Wu, Chang-qing Liu, Xiao-qi Zeng, An-na Jia, Xiao-rong Yin

**Affiliations:** 1 Department of Liver Surgery, West China Hospital / West China School of Nursing, Sichuan University, No. 37, Guoxue Alley, Wuhou District, Chengdu, Sichuan 610041 China; 2 West China School of Nursing / Operating Room of Anesthesia Surgery Center, West China Hospital, Sichuan University, No. 37, Guoxue Alley, Wuhou District, Chengdu, Sichuan 610041 China; 3 Department of Anesthesiology, Anesthesia Surgery Center of West China Hospital / West China School of Nursing, Sichuan University, No. 37, Guoxue Alley, Wuhou District, Chengdu, Sichuan 610041 China

**Keywords:** Tonsillectomy, Recovery from general anesthesia, Oral fluid, Safety, Feasibility

## Abstract

**Background:**

The feasibility and safety of administrating a small amount of oral fluid to children in the early recovery period following tonsillectomy under general anesthesia to reduce the thirst and its associated restlessness remain unknown.

**Methods:**

This study was approved by the institutional ethics committee and adhered to the CONSORT guidelines. Pediatric patients undergoing tonsillectomy who met the inclusion and exclusion criteria of our study were randomized into the study and control groups. In the study group, patients were given a small amount of water instantly after recovering from general anesthesia, which included the recovery of the cough and deglutition reflex, and attaining grade V of muscle strength. The control group was given a small amount of water at 4 to 6 h after the operation. The incidence of nausea and vomiting and the degree of thirst relief were measured and compared between the two groups.

**Results:**

Three hundred patients were randomized into each group. There was no significant difference in the incidence of nausea and vomiting at 20 min after drinking water between the two groups (*P* > 0.05). The thirst score of children over 5 years old in the study group was significantly lower than that of the control group (*P* < 0.05).

**Conclusion:**

Early administration of a small amount of oral fluid to children following tonsillectomy and recovering from general anesthesia is not only safe but also effective in reducing postoperative thirst.

**Trial registration:**

Current Controlled Trials ChiCTR1800020058, 12-12-2018.

## Background

Surgery is currently the main method to treat various diseases, but it is also a stress response for patients, such as preoperative fasting and deprived of drinking, surgical trauma, postoperative anesthesia recovery, etc. Therefore, perioperative nursing is of vital importance for postoperative rehabilitation of patients [1]. Traditionally, in order to avoid adverse events such as nausea, vomiting and coughing caused by the residual effects of anesthetics after general anesthesia, postoperative patients are routinely prohibited from drinking for 4–6 h [2]. Study [3] showed intraoperative anesthetic such as fentanyl and remifentanil would delay the time of oral intake. Some studies [4–7] have shown that oral fluid in the early postoperative period has great benefit to patients undergoing general anesthesia, which include rapid return to normal diet, reduced thirsty, early bowl movement, early ambulation and increased satisfaction. As the enhanced recovery after surgery model has been popularized in recent years, oral intake could be administrated 1–2 h after awake from anesthesia for children undergoing minor surgery [4] and minimizing discontinuation of oral hydration for all patients has been encouraged [8]. Our previous studies [5–7] reported that earlier than 2 h in the postoperative period, even within 20 min of waking from anesthesia, oral hydration did not increase the occurrence of postoperative nausea and vomiting or inhibit gastric peristalsis in the children and adult patients undergoing non-gastrointestinal surgery except tonsillectomy under general anesthesia, which is safe and well tolerated. Not only that, it has become accepted that early oral hydration in the postoperative period could promote early recovery and prevent postoperative ileus [9].

In our setting, tonsillectomy is a solution to a series of symptoms in pediatric patients, such as sleep-disordered breathing caused by tonsillar hypertrophy, obstruction of normal breathing, and influence on the development of children. Therefore, tonsillectomy is a relatively common procedure in pediatric patients [10, 11]. Since tonsillectomy involves the oropharynx of pediatric patients, postoperative oral hydration may be affected. In addition, our previous studies [6, 7] also showed that patients routinely fasted for more than 15 h before surgery, and more than 81% of the children returned to the Post-Anesthesia Care Unit (PACU) after surgery were thirsty and wanted to have oral fluid. The recovery period following general anesthesia is a period for children to fully regain their important physiological functions but is also a period associated with a high risk of adverse events as a result of restlessness [12, 13], which is not uncommon in children and adolescents during the recovery period following general anesthesia, especially among male children [14]. Thirst is common in children undergoing tonsillectomy and often causes severe agitation in the recovery period following anesthesia, leading to increased oxygen consumption, arrhythmia, wound bleeding, etc. In severe cases, patients may inadvertently pull out the infusion tube, urinary catheter, and other medical equipment, which will adversely affect the postoperative recovery [15].

It is well known that children have a higher proportion of water in their bodies than adults, so postoperative fluid management is more important for the pediatrics’ patients. Relevant clinically research is necessary, which should be carried out to explore and consider giving a small amount of oral fluid to children in the recovery period following tonsillectomy under general anesthesia to reduce thirst and its associated restlessness. However, there is little known about the benefit and timing of oral intake for the pediatric patients undergoing tonsillectomy in PACU, and it remains uncertain whether early oral hydration in the PACU is possible after tonsillectomy. Hence, our study aimed to examine the feasibility and safety of early administration of oral fluid and to determine if such intervention would provide thirst relief in this group of patients. The impact of this policy on the primary outcome (thirst scale score) and secondary outcomes (complications including cough, aspiration, nausea, and vomiting) have been observed.

## Methods

### Design

We conducted a prospective, randomized, controlled trial to examine the feasibility and safety of early administration of oral fluid and to determine if such intervention would provide thirst relief in the pediatric patients undergoing tonsillectomy.

This study was approved by the ethics committee of our institution (2017 Review No. 231) with the registration number ChiCTR1800020058 (http://www.chictr.org.cn/showproj.aspx?proj=33847, registered on the 12th December 2018) and adhered to the CONSORT guidelines. We interviewed patients and their parents and then explained the study protocols on the day before surgery. Written informed consent obtained from the parents of all participating children in the surgery day.

### Study population and setting

**Fig. 1:**
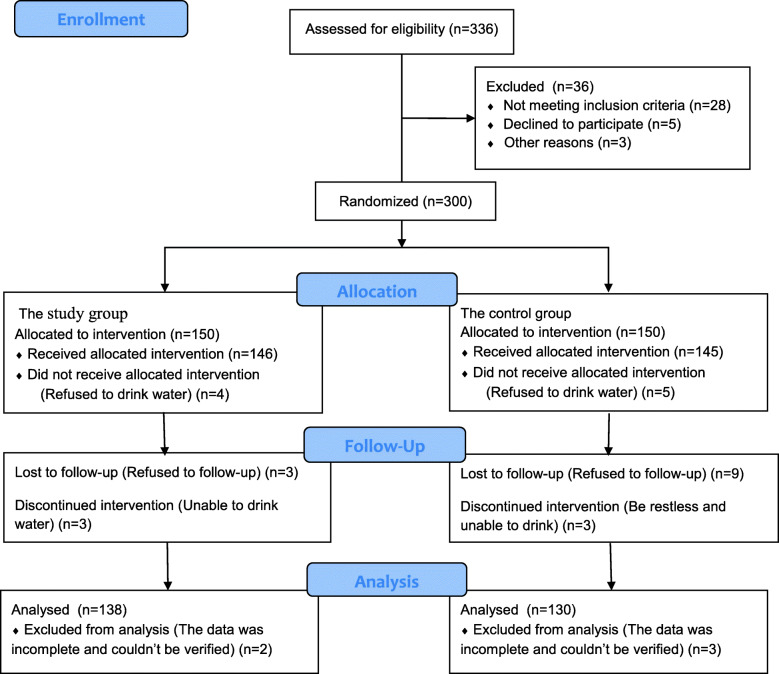
CONSORT 2010 Flow Diagram

In this study, the relief rate of thirst administrated oral fluid in PACU after surgery was selected as an important indicator for the calculation of the sample size. Our previous studies [5–7] showed that the thirsty proportion about 81% of children without drinking water after recovering from anesthesia in PACU. The preset thirst relief rate was 64%, which was less than 71% of the previous studies (after 20 min a small amount of water has been administrated immediately to the patients when postoperative recovering from anesthesia). Considering that the rate of loss of follow-up was about 20%, the sample size was calculated by comparing rates of independent two groups sample. The calculated sample size was 126 cases, that was, at least 126 children were included in each of the study group and control group as the research objects. A total of 300 children undergoing tonsillectomy in the West China Hospital of Sichuan University were recruited and randomized into 2 groups by using the computer-generated random number list: the study group (*n* = 150) and the control group (*n* = 150). In the study group, patients were given a small amount of water instantly after recovering from general anesthesia whereas in the control group, patients were given a small amount of water at 4 to 6 h after recovering from general anesthesia. The number of patients express Fig. 1. Randomization, treatment, and inclusion in analysis.

### Study protocol

When all patients were sent to PACU after their surgery, standard monitoring including electrocardiograph, noninvasive blood pressure monitoring, pulse oximeter, capnograph were applied by trained PACU anesthesiologists and nurses. The administration of oral fluid was introduced in stages. In the study group, when children regained full consciousness following general anesthesia, they were assessed instantly on several criteria: the recovery of coughing and swallowing reflex, no nausea and vomiting, muscle strength returned to level V, stable vital signs, and had a desire for water consumption. Once these criteria were fulfilled, the procedures for oral fluid (water) administration would then be carried out. First, the child’s lips and mouth would be wet with 1–5 ml water. Then, the head of the child would be turned to one side and the child would be given water to drink by a fixed systemically trained anaesthesia monitoring nurse. Depending on the condition of the child, drinking through a straw or with a syringe containing a small amount of water to be injected into the mouth several times. The child would be observed closely for any discomfort such as choking or coughing. If there was no discomfort, the remaining water would then be injected slowly into the mouth, or the child would be encouraged to drink him/herself. Total water volume was restricted to 1.5 mL/kg. After this single fluid intake patients in the study group received no additional fluid until 4–6 h after recovering from general anesthesia, by which time patients had been transferred from the PACU to the ward (to allow ward nurses to be blinded to patient group). In the control group, similar criteria were assessed before children were allowed to drink a small amount of water at 4 to 6 h after recovering from general anesthesia, as usual. All patients received intravenous fluids.

**The inclusion criteria were: **Children aged 3–12 years old undergoing elective tonsillectomy; American Society of Anesthesiologists (ASA) class I-III; Fulfilled the criteria for oral fluid administration as outlined above; Analgesics were administered over 20 min.

**The exclusion criteria were**: Sick children with bleeding, dysphagia, nausea, and vomiting, or needing active resuscitation in PACU.

### Measurements

Primary outcomes was thirst relief, which assessed by thirst scale score using the self-rating thirst scale, consisted of the 0-100 mm verbal numeric scale (0 = not thirsty to 100 = very thirsty) according to patients’ thirst level [5–7]. Secondary outcomes were the complications including cough, aspiration, nausea and vomiting. After drinking water, patients were observed closely for at least 20 min for complications including cough, aspiration, nausea, and vomiting, then thirst relief was assessed and recorded. The number of patients expressing a desire to drink water was also obtained immediately after recovery from general anesthesia and 20 min later. These patients were observed in the PACU for 2 h and then sent to the ward. On admission to the ward, the ward surgeons and nurses were blinded to the policy. When the patients return to the ward, drinking water is done as usual.

### Statistical analysis

SPSS 23.0 was used for statistical analysis. The chi-square test and Fisher’s test were performed to analyze categorical data, whereas the Student’s t-Test was used for continuous data. A *P*-value of < 0.05 was considered statistically significant.

## Results

### Patient characteristics between the two groups

A total of 300 patients were recruited into our study for randomization. However, 15 of these refused to drink water, 12 were lost to follow-up, and 5 had incomplete data collection which could not be verified. Therefore, 268 cases were included in the analyses. Of these, 138 patients were in the study group and 130 were in the control group (Fig. 1), with the age ranging from 3 to 12 years old. The amount of water consumed was less than 1 ml/ kg body weight, approximately 13.56 ± 19.82 ml in the study group. Patients characteristics were compared (Table 1), which showed no statistical difference (*P* > 0.05) between the study and the control groups.

**Table 1:** Comparison of patients characteristics between the study and control groups

**Items**	**Study group**	**Control group**	***P*-value**
	***n* = 138**	***n* = 130**	
**Age **(year), mean ± SD	5.76 ± 2.09	5.87 ± 1.96	0.663
**Gender **(n,%)			0.807
Male	73 (52.9)	71 (54.6)	
Female	65 (47.1)	59 (45.4)	
**Weight **(Kg), mean ± SD	22.11 ± 8.92	22.44 ± 7.74	0.751
**Preoperative fasting time **(h), mean ± SD	10.34 ± 2.57	10.43 ± 2.56	0.765
**Anesthesia time **(min), mean ± SD	68.33 ± 22.80	66.73 ± 21.83	0.558
**Time of operation **(min), mean ± SD	34.79 ± 14.22	34.97 ± 17.42	0.926
**Intraoperative infusion volume **(ml), mean ± SD	190.25 ± 120.79	188.58 ± 134.53	0.914
**ASA classification **(n,%)			0.966
I	7 (5.1)	5 (3.8)	
II	127 (92.0)	123 (94.6)	
III	4 (2.9)	2 (1.5)	

### Comparison of perioperative anesthetic drugs usage between the two groups

The use of atracurium was significantly higher in the control group compared with the study group (*P* = 0.02). There was no other statistically significant difference between the two groups in the use of perioperative anesthetic drugs (Table 2).

**Table 2:** Comparison of the anesthetic drugs usage between two groups

**Items**	**Study group (*N* = 138)**	**Control group (*N* = 130)**	***P*-value**
Fentanyl (n,%)	122 (88.4)	113 (86.9)	0.715
Sufentanil (n,%)	13 (9.4)	20 (15.4)	0.192
Remifentanil (n,%)	24 (17.4)	30 (23.1)	0.287
Sevoflurane (n,%)	137 (99.3)	126 (96.9)	0.202
Atracurium (n,%)	111 (80.4)	118 (90.8)	0.023
Propofol (n,%)	127 (92)	115 (88.5)	0.410
Dexmedetomidine (n,%)	80 (58.0)	67 (51.5)	0.326
Atropine (n,%)	80 (58.0)	66 (50.8)	0.270
Tramadol (n,%)	56 (40.6)	59 (45.4)	0.460
Lidocaine (n,%)	9 (6.5)	15 (11.5)	0.199
Ondansetron hydrochloride tablets (n,%)	120 (87.0)	122 (93.8)	0.065
Midazolam (n,%)	120 (87.0)	106 (81.5)	0.243
Granisetron (n,%)	5 (3.6)	5 (3.8)	1.000
Dexamethasone (n,%)	102 (73.9)	96 (73.8)	1.000
Neostigmine (n,%)	6 (4.3)	3 (2.3)	0.502
Hemocoagulase Bothrops Atrox for Injection (n,%)	46 (33.3)	43 (33.1)	1.000
Penehyclidine (n,%)	2 (1.4)	2 (1.5)	1.000

### Comparison of the thirst relief rate and thirst scores between the two groups

In the study, we found that children under 5 years old were not very good at thirst score, so children aged 3–5 years old were not included in the thirst score analysis. The thirst relief rate was analyzed statistically in all the children. Before oral fluid was administered, the thirst rate of patients in the study group was 91.3%(126/138) and that of patients in the control group was 93.1%(121/130), with no statistically significant difference (χ^2^ = 0.291,*P* = 0.589). However, after 20 min of oral fluid was administered, the thirst rate of patients in the study group was 41.3% (57/138) and that of patients in the control group was 87.7%(114/130), the difference was statistically significant (χ^2^ = 62.37,*P* < 0.001). There were 189 children over 5 years old in our study, and there was no significant difference (*P* > 0.05) in the thirst score before oral fluid was given in the PACU. However, the thirst score in the study group after 20 min of oral fluid consumption in the PACU was significantly lower compared with the control group (*P* = 0.001), (Showed in Table 3).

**Table 3:** Comparison of the thirst relief rate and thirst scores between the two groups

**Items**	**Study group (*N* = 138)**	**Control group (*N* = 130)**	***P*-value**
Thirst relief rate before oral fluid was administered (n, %)	126 (91.3%)	121 (93.1%)	0.589
Thirst relief rate of 20 min after oral fluid was administered (n, %)	57 (41.3%)	114 (87.7%)	< 0.001
Thirst scales just after recovery from anesthesia (*n* = 92), mean ± SD	46.85 ± 40.43	43.20 ± 35.72	0.660
Thirst scales 20 min after oral fluid was administered at PACU (*n* = 97), mean ± SD	36.52 ± 36.30	54.64 ± 34.79	0.001

### Comparison of the incidence of nausea and vomiting between the two groups

There was no incidence of aspiration in this study. There was no significant difference in the incidence of nausea and vomiting between the two groups before and after oral fluid consumption at the PACU, or in the ward (*P* > 0.05). However, we observed incidences of nausea and vomiting occurred in the control group in the ward, although this difference was not statistically significant (*P* = 0.054) (Table 4).

**Table 4:** Comparison of incidence of nausea and vomiting between two groups

**Items**	**Study group (*N* = 138)**	**Control group (*N* = 130)**	***P*-value**
**Incidence of nausea at PACU**, n(%)			
Before drinking at the PACU	0	2 (1.5)	0.234
20 min after drinking at PACU	2 (1.4)	3 (2.3)	0.676
**Incidence of vomiting at PACU**, n(%)			
Before drinking at the PACU	0	0	
20 min after drinking at PACU	1 (0.7)	1 (0.8)	1.000
**Nausea and vomiting before returning to the ward for food**, n (%)	0	1 (0.8)	0.485
**Nausea after eating in the ward**, n (%)	0	4 (3.1)	0.054
**Vomiting after eating in the ward**, n (%)	0	3 (2.3)	0.113
**Nausea and vomiting occurred 24 h after surgery**	3 (2.2)	2 (1.5)	1.000

## Discussion

The finding of this prospective randomized controlled study showed that early oral fluid for pediatric patients immediately after undergoing tonsillectomy under general anesthesia, was not only safe and well tolerated, but also significantly relieved patients’ thirst than delayed oral hydration.

Pediatric patients undergoing tonsillectomy are usually allowed to start oral intake gradually at 6 h after recovering from general anesthesia. The types of oral intake include ice drinks, such as ice milk and pure ice cream without impurities which help to relieve pain and reduce the risk of bleeding. Fruit and juice are advised to avoid because these contain fruit acids, which may cause pain and affect wound healing by stimulating the wound [16]. After tonsillectomy, children are usually advised to stay quietly in bed, speak little, and avoid coughing forcefully to prevent bleeding. Study advocate that children should drink cold fluid on the day after surgery [17]. In our study, mineral water at normal room temperature was given to the children in the early phase of recovery from general anesthesia in the study group following tonsillectomy. We did not observe any adverse events such as bleeding and there was no increase in the incidence of nausea or vomiting. Our data suggested that early administration of oral fluid after general anesthesia for tonsillectomy is safe.

The most common symptoms after tonsillectomy are wound pain and pain upon swallowing. Some children also experience nausea and vomiting. The routine use of intravenous dexamethasone in this setting has been reported to reduce the incidence of vomiting from 27–11% [18]. In our study, 73.8% of the children received dexamethasone intraoperatively, and the incidence of nausea and vomiting after the administration of oral fluid following general anesthesia was only 0.7%. Such a low incidence of nausea and vomiting in our study may be related to the intraoperative use of dexamethasone and antiemetic drugs.

The study by Chen et al. [19] reported that the time of eating after general anesthesia for non-abdominal surgery should be determined by the extend of the operation, the method of anesthesia, and the children’s reaction. For the body surface or limb surgery, the body reaction is relatively mild. Patients may not eat if they are still under the influence of anesthesia with nausea and vomiting. Hence, patients usually eat when they have fully recovered from anesthesia that their nausea and vomiting have subsided. In the ambulatory surgical setting, a study [20] has demonstrated no statistically significant difference in the incidence of nausea and vomiting within 24 h after minor surgery between the drinking and the non-drinking group. Another study [21] has shown that among pediatric patients undergoing adenotonsillectomy, there was a significantly higher incidence of emesis when patients were encouraged to drink compared to patients who drank voluntarily, with the incidence of emesis escalated in both groups when the target volume of 240 ml was reached. Further study has also reported that early oral fluid administration in adult patients undergoing non-gastrointestinal surgery was safe with patients experiencing lower scales in thirst and oropharyngeal discomfort [5], and early postoperative oral hydration in small quantity in children was feasible without increasing the incidence of nausea, vomiting, choking, and aspiration [22]. Our study has further emphasized that early administration of oral fluid is feasible, safe, and associated with relived thirst and reduced incidence of postoperative nausea and vomiting in children who has the desire to drink. Such feasibility and safety observed were most likely attributable to our stringent criteria in patient assessment before allowing oral fluid in addition to close supervision and observation of fluid intake in the early phase.

There were some limitations to this study. We did not explore the optimal amount of fluids allowed after tonsillectomy. Furthermore, there was no blinding in the assessment of nausea/vomiting. Moreover, the long-term efficacy of a small amount of oral fluid for children undergoing tonsillectomy has not been determined, which warrants further studies.

## Conclusions

In conclusion, the early administration of a small amount of oral fluid in children undergoing tonsil surgery and recovering from general anesthesia is not only safe but also effective in reducing postoperative thirst. The recovery of cough and swallowing reflex and muscle strength must be determined to ensure the safety of such practice.

## Data Availability

The data that supports the findings of this study are available on request from the corresponding author. The data are not publicly available due to privacy or ethical restrictions.

## Abbreviations

ASA: American Society of Anesthesiologists
PACU: Post-Anesthesia Care Unit
